# Insight into antiphospholipid syndrome: the role and clinical utility of neutrophils extracellular traps formation

**DOI:** 10.1186/s12959-024-00598-4

**Published:** 2024-03-28

**Authors:** Shams ElDoha Galal ElDin Zaiema, Menna Allah Zakaria Mohammad Ali Abou Elwafa, Shaymaa Gamal Arafa Hassan, Radwa Hassan Abou El Fotoh El Adwey, Raghda Mohammed Mostafa Ghorab, Raghda El Sayed Abdel Monem Galal

**Affiliations:** 1 Department of Clinical and Chemical Pathology, Ain shams University, Faculty of medicine, Cairo, Egypt; 2 Department of Rheumatology-Internal Medicine, Ain shams University, Faculty of medicine, Cairo, Egypt; 3 Department of Immunology-Internal Medicine, Ain shams University, Faculty of medicine, Cairo, Egypt; 4 Immunogenetics Department, Human Genetics and Genome Research Institute, National Research Centre, Cairo, Egypt

**Keywords:** Antiphospholipid syndrome, Antiphospholipid antibodies, NETosis, Myeloperoxidase, Histones, Thrombosis, Cutoff for NETs activation, Anti-NETosis therapy/NETs inhibitors

## Abstract

Antiphospholipid syndrome (APLS) is a systemic immune dysregulation distinguished by repetitive complications and pregnancy loss in the absence of definite etiology. Most research focuses on the laboratory detection and clinical features of APLS, but its precise etiology remains to be deeply explored. NETosis is a newly developed theory in the pathophysiology of APLS which may serve as the missing bridge between coagulation and inflammation reaching the disease progression and severity. We aimed in this study to navigate the prognostic role of NETosis in thrombotic APLS. Our study included 49 newly diagnosed APLS patients (both 1ry and 2ry) who met clinical and laboratory criteria as per *the international consensus statement on the update of the classification criteria for definite APLS* and were sub-classified according to the occurrence of thrombotic events in thrombotic and non-thrombotic types. In addition, 20 sex and age-matched reactive subjects and 20 sex and age-matched healthy volunteer controls were enrolled. NETosis formation was assessed by measuring serum Myeloperoxidase (MPO) and Histones level using the enzyme-linked immunosorbent assay (ELISA) technique. Both MPO and Histones levels were able to discriminate among APLS cases from normal controls, showing significant cutoffs of > 2.09 ng/ml for MPO and > 1.45 ng/ml for Histones (AUC values were 0.987and 1.000, respectively). These values can be used as predictors for NETosis pathophysiology in APLS patients. Additionally, these markers demonstrated a significant association with several prognostic indicators, including thrombosis, higher PT and INR, and lower hemoglobin (Hb) levels which are supposed to be ameliorated by using NETs inhibitors. *In conclusion*, we suggest that measuring NETosis markers, MPO, and Histones, in the early course of APLS using proposed cutoff values will facilitate the timely initiation of anti-NETosis therapy and improve the overall prognosis, particularly for patients with thrombotic APLS.

## Introduction

Antiphospholipid syndrome (APLS) is a thrombo-inflammatory defect, distinguished by vascular thrombosis and/or obstetric complications. It is driven by the presence of circulating antiphospholipid (aPL) antibodies, namely anticardiolipin antibodies (ACL), anti-β2glycoprotein I antibodies (B2GP1), and lupus anticoagulant (LAC) [1].

Newly published population-based studies have estimated the epidemiology of APLS, with an incidence ranging from 1 and 2 cases per 100,000 and a prevalence ranging from 40 and 50 cases per 100,000. The prevalence of aPL in patients with obstetric complications was 6–9%, in vascular thrombosis was 9–10% and the mortality rate of patients with APLS estimated higher about 50–80% when compared to the general population [2].

APLS either exists as an isolated disorder (primary APLS), or secondary to another autoimmune condition, especially systemic lupus erythromatosis (SLE) as it complicates about one-third of SLE cases worldwide [3]. In Egypt, a large study was conducted on adult patients with SLE and found that APLS was reported in 17.1% of them [4].

As per *the international consensus statement on the update of the classification criteria for definite APLS*, thrombotic APLS is diagnosed based on the presence of persistently positive aPL antibodies and at least one clinical finding that indicates vascular thrombosis or pregnancy morbidity [5]. However, beyond vascular thrombosis and/or obstetric complications, there are non-criteria manifestations of APLS such as thrombocytopenia, autoimmune hemolytic anemia, renal dysfunction, cardiac valve disease, cognitive impairment, skin ulcers, or pulmonary hemorrhage which can happen even with optimum administration of anticoagulation therapy [6].

The solid dependency on aPL antibodies to confirm or exclude the diagnosis of APLS has unfortunately been hindered by the multifactorial pitfalls that affect the aPL profiles assessment. Currently used laboratory methods can be perplexed by many challenges such as anti-thrombotic treatment and inflammation besides, some patients may only produce these antibodies intermittently. Consequently, misinterpretation or overreliance on aPL results alone can lead to improper evaluation and management for APLS patients which continue to be challenging for clinicians [7–9].

Recently, newly announced research has highlighted the important role of neutrophil activation and the release of neutrophil extracellular traps (NETs) in a process known as NETosis, a special form of programmed cell death, which is thought to serve as the basis of immune-mediated thrombosis in APLS [10]. It is characterized by the release of neutrophil granule components such as neutrophils elastase (ELANE) and myeloperoxidase (MPO) into the cytosol, together with chromatin de-condensation and histones modification as a result of interaction between inflammation with immune dysregulation. These proteins when come in contact with the bloodstream, they result in vascular inflammation, coagulopathy, and platelet activation which eventually leads to thrombosis [11].

Whether APLS-related thrombosis is clinically attributed to these pathological mechanisms of NETosis is not fully proven however, the interaction between coagulation and inflammation alongside the disease progression cannot be solely explained by aPL antibodies and still needs more explanation to be fully understood [11]. Besides, the promising development of NETosis-targeted therapy such as toll-like receptor inhibitors and reactive oxygen species scavengers, can prevent uncontrolled NET development [12].

In this research, we aimed to study the role and clinical utility of NETosis in newly diagnosed APLS patients especially thrombotic type concerning other clinical and laboratory indicators.

## Patients and methods

### Patients

This is a case-control study that has been performed on APLS patients. All patients were recruited from Ain Shams University Rheumatology and Immunology departments of Internal Medicine Hospital, Cairo, Egypt. Patients with ages < 14 and > 80 years old, other causes of thrombophilia (inherited or acquired), malignancies (hematological or non-hematological) were excluded. Eligible 49 newly diagnosed APLS patients who met the clinical and laboratory criteria according to *the international consensus statement on the update of the classification criteria for definite APLS* [5] were recruited into the study. The presence of thrombotic events (deep venous thrombosis, Obstetric complication, and stroke) was used to subcategorize APLS patients into the Thrombotic APLS group (*n* = 35) and Non-thrombotic APLS group (*n* = 14), and according to the underlying etiology into1ry APLS (*n* = 26) and APLS 2ry to SLE group (*n* = 23). Finally, they were sub-classified according to the number of positive aPL antibodies (LAC, and IgG or IgM classes for ACL and B2GB1) to study the strength of autoimmune-mediated attacks present in APLS patients, single positivity (LAC only) group (*n* = 10), double positivity (LAC, B2GB1or ACL) group (*n* = 9), and triple positivity (LAC, B2GB1, ACL) group (*n* = 30) (Table 1). In addition, 20 age- and sex-matched reactive subjects with reactive leucocytosis (WBC count > 20x10^3^μL with high Neutrophils/lymphocytes (N/L)) ratio were enrolled from Ain Shams Hospital for causes other than an autoimmune disorder or thrombophilia screen such as post-operative, infection or other inflammatory condition. Moreover, 20 age- and sex-matched healthy volunteer subjects were enrolled as a control group. All data concerning each patient’s disease history was collected from their hospital files.

**Table 1:** Characteristics of APLS patients’ group

	APLS Group
	No. = 49
Thrombotic	
No	14 (28.6%)
Yes	35 (71.4%)
1ry/2ry APLS	
1ry	26 (53.1%)
2ry	23 (46.9%)
Number of lab Positive Autoantibodies	
Single positivity	10 (20.4%)
Double positivity	9 (18.4%)
Triple positivity	30 (61.2%)

### Methods

- Sampling Method: A peripheral blood sample of 10 cm was collected from each subject to obtain a set of four blood tubes (on EDTA, sodium citrate 3.2%, and two anticoagulant-free tubes).
- *All participants included in this study were subjected to the following procedures:*
  1. Complete blood count using EDTA blood samples on Coulter GenS system 2 (Beckman-Coulter, Miami, FL)) and microscopic examination of Leishman-stained peripheral blood (PB) smear with special focus on WBCs (including absolute N/L ratio) and platelets count.
  2. Citrated blood tube was separated by centrifugation at 3000×g for 15 min, and plasma was used for assessing full coagulation profile including PT, INR, PTT, and LAC (diluted Russell Viper Venom Time (dRVVT) based methods).
  3. Non-fasting venous blood samples were collected on an anticoagulant-free tube and separated by centrifugation at 1000×g for 15 min, and their serum was used for ESR, and CRP. Assessment of ACL (IgG and IgM) and Anti-B2GBI (IgG and IgM) were done only for APLS patients using ELISA (since the aPL Ab could be non-significantly present in some reactive conditions or in normal people which may cause unnecessary bias of other significant data).
  4. Another non-fasting venous blood sample was collected on an anticoagulant-free tube and separated by centrifugation at 1000×g for 15 min, and their serum aliquots were stored at − 20 °C within 1 h of collection and used for assessment of markers of NETosis formation using sandwich ELISA KIT, Human Myeloperoxidase, MPO (Bioassay Technology Laboratory, E0880Hu, China) and Human Histone-H3 (Bioassay Technology Laboratory, E5419Hu, China), assays were done according to manufacturer’s instruction.

## Statistical methods

The Statistical Package for Social Science (IBM SPSS version 23) was used in this research to analyze collected data. The quantitative data was shown as means, standard deviations, and ranges when they displayed a parametric distribution, and as medians with interquartile ranges (IQR) when they displayed a non-parametric distribution. For qualitative data, they were shown as percentages and numbers. The groups were compared using the Chi-square test and/or Fisher exact test. To compare 2 different groups, the independent t-test was used with quantitative data and parametric distribution, whereas the Mann-Whitney test was used for non-parametric distribution. The receiver operating characteristic curve (ROC) was used to identify the optimal cut-off point and its sensitivity, specificity, positive predictive value, negative predictive value, and area under the curve (AUC). The allowable margin of error was set at 5%, while the confidence interval was set at 95%. As a result, the *P*-value was considered significant at the level of 0.05.

## Results

The APLS patients’ group were 49 with ages ranging from 17 to 53 years (mean 34.86 years) were enrolled in this study with a female: male ratio of 1.4:1. In addition, 20 age- and sex-matched reactive subjects were enrolled and 20 age- and sex-matched healthy volunteer were also included in most of the laboratory assessments.

### Comparison between the studied groups regarding demographic and laboratory parameters

Many studied parameters showed a substantial difference in the APLS group in comparison to healthy controls and the reactive group. The APLS cases had a highly significant lower Hb and PLT level (*P*-value < 0.01) when compared to healthy controls, they also showed a significantly higher N/L ratio (*P*-value < 0.05), and a highly significant elevated INR, PTT, ESR and CRP (*P*-value < 0.01). In comparing APLS patients with the reactive group, there was a non-significant difference regarding Hb level but there was a highly significant decrease in PLT count (*P*-value < 0.01). On the other hand, the reactive group showed a highly significant rise in WBC count, neutrophils count, N/L ratio, and CRP (*P*-value < 0.01) with a significant increase in PT (*P*-value < 0.05) when compared to APLS patients. ESR showed a highly significant rise in APLS patients (*P*-value < 0.01) when compared with the reactive group.

### Studying the value of MPO and histones combination in all studied groups

**Fig. 1:**
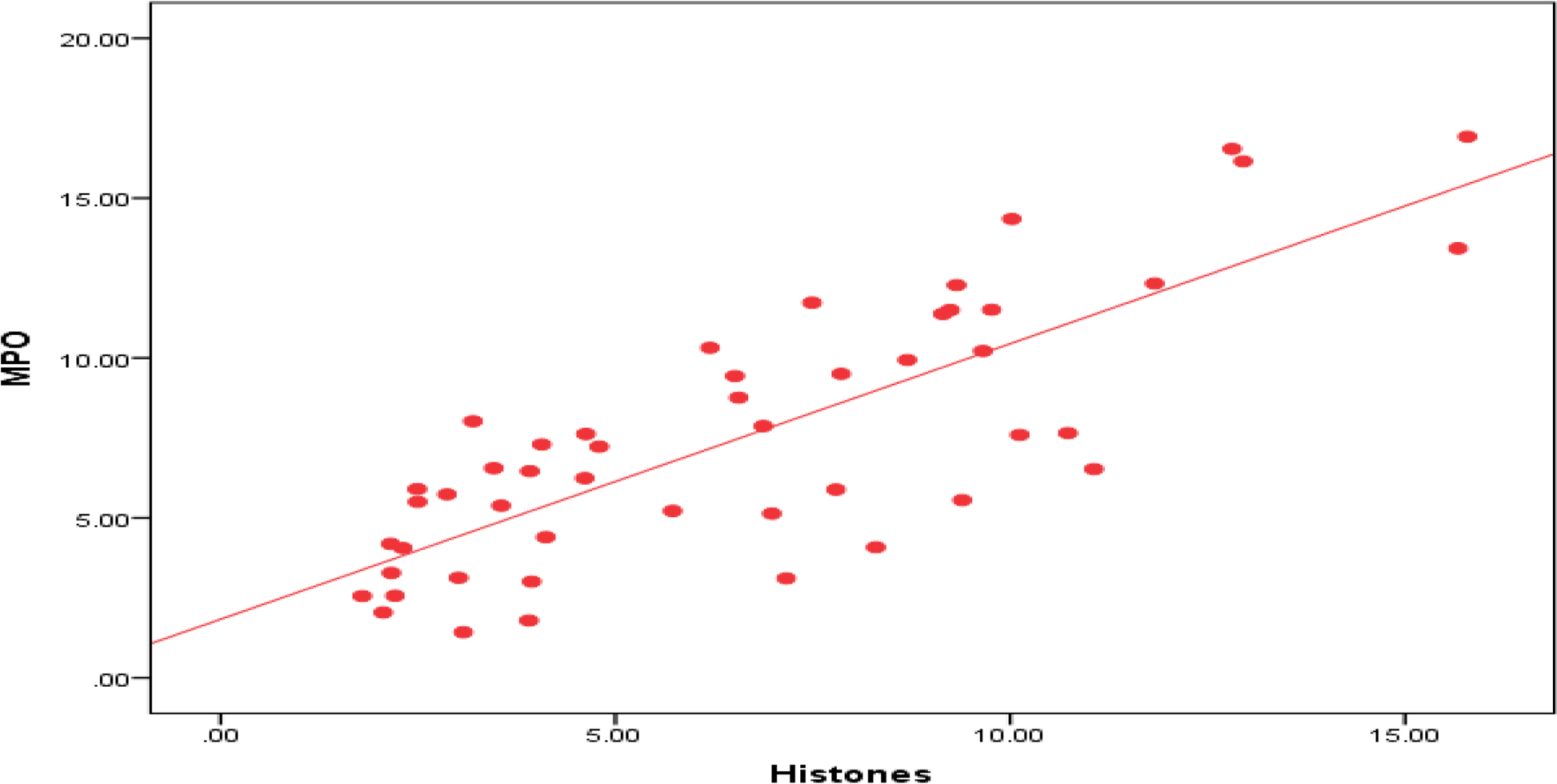
Correlation of MPO and Histones levels in all studied groups

The human MPO and Histones levels showed a highly significant correlation in all studied groups (APLS, reactive group, and normal controls) and supporting the neutrophils MPO-DNA complex formation and NETs activation (Fig. 1). The MPO and Histones levels showed a highly significant elevation in the studied APLS and the reactive groups compared to controls (*P*-value < 0.01). The MPO and Histones levels were comparable with no significant difference between APLS patients and the reactive group (*P*-value > 0.05).

Cut-off values were determined for the MPO and Histones to predict threshold levels that indicate activation of NETosis process. According to our results, in both APLS patients and the reactive group, Histones at a cutoff > 1.45 ng/ml were the best predictor for active NETosis (AUC was 1.00; with a sensitivity of 100 and a specificity of 100). MPO at a cutoff > 2.09 ng/ml was acknowledged as the best predictor for active NETosis in APLS patients (AUC was 0.987; with a sensitivity of 93.88 and a specificity of 97.90) (Table 2). In the reactive group, MPO at a cutoff > 3.62 ng/ml was identified as the best predictor for active NETosis (AUC was 0.990; with a sensitivity of 95.0 and a specificity of 100.0) (Table 3).

**Table 2:** AUC, sensitivity, specificity, and Cut-off value of MPO and histones that differentiate between APLS and control group

	Cut-off point	AUC	Sensitivity	Specificity	PPV	NPV
MPO (ng/ml)	> 2.09	0.987	93.88	95.00	97.90	86.40
Histones (ng/ml)	> 1.45	1.000	100.00	100.00	100.00	100.00

**Table 3:** AUC, sensitivity, specificity, and Cut-off value of MPO and histones that differentiate between the Reactive group and the control group

	Cut-off point	AUC	Sensitivity	Specificity	PPV	NPV
MPO (ng/ml)	> 3.62	0.990	95.0	100.0	100.0	95.2
Histones (ng/ml)	> 1.45	1.000	100.0	100.0	100.0	100.0

### Correlation studies of MPO level with the other studied parameters in the normal control group, reactive group, and APLS patients’ group

In all studied groups, MPO levels showed a significant positive correlation with WBC count, neutrophils count, and PT level (*P*-value < 0.05), and a highly significant positive correlation with INR and ESR (*P*-value < 0.01), Meanwhile, there was a high significant negative correlation found between MPO and Hb level (*P*-value < 0.01). In APLS patients there was a highly significant positive correlation found between MPO and PT (*P*-value < 0.01), a significant positive correlation with INR (*P*-value < 0.05), and a highly significant negative correlation with CRP (*P*-value < 0.01). In the reactive group, MPO showed a highly significant positive correlation with Histones level (*P*-value < 0.01) (Table 4).

**Table 4:** Correlation studies of MPO level with the other studied parameters in all studied groups, normal control group, reactive group, and APLS patients’ group

	All studied groups		Control group		Reactive Group		APLS	
	*r*	*P*-value	*r*	*P*-value	*r*	*P*-value	*r*	*P*-value
Age (years)	0.177	0.098	0.054	0.820	0.009	0.971	0.234	0.105
HB g/dL	**−0.352**	0.001**	−0.064	0.789	− 0.061	0.799	0.181	0.213
WBCs (×10^3^/ul)	**0.230**	0.030*	0.292	0.211	0.031	0.898	0.120	0.410
NE (×10^3^/ul)	**0.226**	0.034*	0.338	0.144	0.011	0.962	0.041	0.780
N/L ratio	0.164	0.125	0.293	0.210	−0.013	0.956	−0.129	0.377
PLT (×10^3^/ul)	−0.189	0.076	0.232	0.324	−0.160	0.500	0.042	0.774
PT (min)	**0.218**	0.040*	0.035	0.883	−0.211	0.373	**0.370**	0.009**
INR	**0.341**	0.001**	0.233	0.322	−0.111	0.651	**0.315**	0.028*
PTT (min)	0.137	0.210	0.108	0.652	−0.246	0.296	−0.084	0.585
CRP (mg/dL)	0.167	0.132	0.287	0.220	−0.297	0.203	**−0.457**	0.002**
ESR (mm/hour)	**0.349**	0.001**	−0.082	0.731	−0.043	0.858	0.119	0.415
**Histones** (ng/ml)	**0.836**	0.000**	0.161	0.497	**0.659**	0.002**	**0.798**	0.000**

*P*-value > 0.05: Non-significant; *P*-value < 0.05*: Significant; *P*-value < 0.01**: Highly significant, analysis was done by Spearman correlation coefficient

### Correlation studies of histones level with the other studied parameters in all studied groups, normal control group, reactive group, and APLS patients’ group

In all studied groups, Histones levels showed a significant positive correlation with PT, INR (*P*-value < 0.05), a highly significant positive correlation with ESR and MPO levels (*P*-value < 0.01), Meanwhile, Histones level showed a highly significant negative correlation with Hb level (*P*-value < 0.01). However, unlike MPO, Histones showed non-significant correlations with WBC count and neutrophils count. In APLS patients, Histones showed significant positive correlations with PT, and INR (*P*-value < 0.05), and a significant negative correlation with CRP (*P*-value < 0.05) (Table 5).

**Table 5:** Correlation studies of Histones level with the other studied parameters in all studied groups, normal control group, reactive group, and APLS patients’ group

	All studied groups		Control group		Reactive Group		APLS	
	*r*	*P*-value	*r*	*P*-value	*r*	*P*-value	*r*	*P*-value
Age (years)	0.069	0.519	− 0.116	0.627	0.104	0.664	−0.011	0.941
HB (g/dL)	−0.371**	**0.000**	−0.387	0.091	−0.244	0.301	0.116	0.428
WBCs (×10^3^/ul)	0.139	0.193	0.104	0.661	−0.041	0.864	0.146	0.317
NE (×10^3^/ul)	0.132	0.217	−0.006	0.981	−0.080	0.738	0.082	0.576
N/L ratio	0.079	0.459	−0.127	0.592	−0.126	0.596	−0.012	0.937
PLTs (×10^3^/ul)	−0.190	0.074	−0.051	0.832	−0.192	0.419	0.212	0.144
PT (min)	0.219*	**0.039**	−0.002	0.994	−0.055	0.818	0.306*	**0.032**
INR	0.248*	**0.020**	0.266	0.256	−0.331	0.167	0.283*	**0.049**
PTT (min)	0.136	0.216	0.295	0.207	−0.062	0.794	−0.159	0.298
CRP (mg/dL)	0.133	0.230	0.189	0.424	−0.064	0.787	−0.320*	**0.036**
ESR mm/hour	0.442**	**0.000**	−0.425	0.062	0.152	0.524	0.242	0.093
MPO (ng/ml)	0.836**	**0.000**	0.161	0.497	0.659**	**0.002**	0.798**	**0.000**

*P*-value > 0.05: Non-significant; *P*-value < 0.05: Significant; *P*-value < 0.01: Highly significant, ‡: analysis was done by Spearman correlation coefficient

### Studying the relation between MPO and histone levels with the clinical characteristics of APLS patients’ group

In APLS patients’ groups, MPO and Histones levels showed a highly significant increase in thrombotic APLS patients (*n* = 35) when compared with non-thrombotic ones (*n* = 14) (*P*-value < 0.01). Also, Histones level showed a highly significant elevation in patients with aPL double positivity (*n* = 9) (LAC, B2GB1or ACL) and triple positivity (*n* = 30) (LAC, B2GB1 and ACL) when compared to those with single positivity (*n* = 10) (LAC only) (*P*-value < 0.01). Meanwhile, MPO levels showed no significant difference among APLS patients regarding their positivity for aPL antibodies. Both MPO and Histones could not differentiate between 1ry (*n* = 26) and 2ry APLS (*n* = 23) (Table 6).

**Table 6:** Relation between MPO and Histones levels with the clinical characteristics of APLS patients’ group

		Histones (ng/ml)		*P*-value
		Median (IQR)	Range	
Thrombotic^a^	No	2.68 (2.16–3.2)	1.79–10.73	0.000**
	Yes	7.79 (4.61–9.76)	2.31–15.79	
1ry/2ry APLS^a^	1ry	7.07 (3.2–9.66)	1.79–15.79	0.42
	2ry	4.61 (3.46–8.7)	2.15–12.95	
Number of lab Positive Autoantibodie^b^	Single positivity	2.4 (2.16–4.07)	2.06–9.76	0.006**
	Double positivity	7.16 (3.46–9.39)	3.07–10.73	
	Triple positivity	7.24 (4.12–9.66)	1.79–15.79	
		MPO (ng/ml)		*P*-value
		Median (IQR)	Range	
Thrombotic^a^	No	3.73 (2.57–5.9)	1.43–8.76	0.001**
	Yes	7.62 (5.56–11.51)	1.79–16.92	
1ry/2ry APLS^a^	1ry	6.21 (4.08–10.22)	2.04–16.92	0.446
	2ry	7.23 (5.39–11.73)	1.43–16.54	
Number of lab Positive Autoantibodies^b^	Single positivity	4.12 (3.13–7.3)	2.04–11.51	0.073**
	Double positivity	7.6 (5.56–8.02)	1.43–9.94	
	Triple positivity	7.43 (5.39–11.73)	1.79–16.92	

*P*-value > 0.05: Non-significant; *P*-value < 0.05*: Significant; *P*-value < 0.01**: Highly significant, ^a^: Mann-Whitney test; ^b^: Kruskall-Wallis’s test

## Discussion

APLS is an immune-inflammatory-thrombotic dysregulation distinguished by repeated thrombosis and provoked obstetric complications mostly rooted in the constant positivity of aPL. Neutrophil activation is considered another important player in the pathophysiology of both SLE and APLS through the formation of net-like structures in a process referred to as NETs formation. Assessment of NETosis-specific markers such as serum levels of MPO-DNA, citrullinated histones, and the neutrophil elastase-DNA complex is becoming essential for initiating NETs inhibitors. Histones proteins are a tectonic portion of chromatin built by DNA which, on the one hand, represent an autoantigen in SLE and APLS and, on the other hand, serve as an indicator for NETosis activation. Insufficient data is available on the clinical role for assessing the MPO and Histones level in APLS, especially the thrombotic type.

Given these data, we designed our study to explore the potential role of MPO and Histones in APLS Egyptian patients. We believe that this study is the first trial to analyze the prognostic effect of this combination in APLS patients.

In our study, we analyzed a group of demographic and laboratory parameters in APLS patients in comparison to normal volunteer subjects. Also, we involved a reactive inflammatory group with a high WBC count (>20x10^3^μL with a high N/L ratio) to monitor the changes that are specific for NETosis activation in APLS since NETs formation can also occur in several other conditions such as malignancy, inflammation, and infection and plays a significant role in the body defense and innate immunity [13].

As expected, the APLS cases showed anemia and thrombocytopenia when compared to healthy control however, they had higher N/L ratio, INR, PTT, ESR, and CRP which is mostly related to the ongoing chronic inflammatory-thrombotic pathology in APLS. In comparing APLS patients with the reactive group, APLS patients showed also lower PLT count and higher ESR levels with no significant difference regarding Hb. However, the reactive group showed higher WBC count, neutrophils count, N/L ratio, and CRP when compared to APLS patients. These findings could be explained by the underlying pathology in each group and the chosen criteria for the reactive subjects. Also, According to Kourilovitch and Galarza–Maldonado, 2023 [14], neutrophil activation and NETs liberation lead to enhanced release of reactive oxygen species (ROS) and MPO that leads to cellular injury followed by chronic inflammation suppressing T- and B-cell immune functions, which may explain the higher N/L ratio in APLS and the reactive group. Furthermore, many studies stated the occurrence of thrombocytopenia and hemolytic anemia in APLS which are now included in the “Non-criteria features” of APLS [15]. Klack et al. 2013 found that APLS patients have a higher incidence of iron deficiency anemia when compared to healthy controls [16].

To demonstrate NETs formation in our studied groups, we explored the levels of MPO and Histones in all studied subject groups. MPO and Histones levels in APLS patients and reactive subjects were significantly higher than in the normal control group. In addition, the MPO and Histones levels were comparable with no significant difference between APLS patients and the reactive group. All these former data provided evidence for NETs formation. This was in agreement with de Moraes Mazetto et al. 2022 who stated that APLS patients especially thrombotic type showed higher serum MPO-DNA complex levels than in healthy controls and that its level correlated with the disease severity and poor outcome [11].

Threshold values were determined in our research for the MPO and Histones levels to predict activation of NETosis process. According to our results, MPO at a cutoff > 2.09 ng/ml was acknowledged as the best predictor for active NETosis in APLS patients which was slightly lower than that in the reactive group, (MPO at a cutoff > 3.62 ng/ml). In both APLS patients and the reactive group, Histones at a cutoff > 1.45 ng/ml were the best predictor for active NETosis. In recent published research, there was no determined cutoff for any of the NETs markers. We believe that determining a specific cutoff will help timely initiation of anti-NETosis therapy with subsequent prevention of unwanted sequelae of NETs formation.

In our work, we also studied the correlation of MPO and Histones levels with the other studied parameters in all studied subjects, normal controls group, reactive group, and APLS patients’ group to determine their prognostic role. In all studied groups, there was a significant positive correlation found between MPO levels, WBC count, and neutrophil count. This correlation was not found with Histones, which could be explained as neutrophils being very rich in MPO forming around 5% of its actual weight and stored in the azurophilic granules which are released upon neutrophils activation with subsequent NETs formation in response to ongoing inflammatory/immune dysregulation [17].

Additionally, in our study, there was a significant negative correlation found between MPO and Hb levels in all studied groups. The presence of anemia during NETs formation in a previous study on canines was found to be mostly attributed to the pathological inflammatory condition, organ damage, and autoimmune hemolytic process present as a part of the pathogenesis of APLS or from bleeding resulting from anticoagulant therapy used in APLS patient to prevent thrombosis [18].

MPO and Histones levels showed a significant negative correlation with CRP which indicated that NETs formation in APLS patients did not occur as an acute phase activation but rather due to another aetiology. In studies on NETs by Bravo-Barrera et al., 2017 [19], they have proved that autoantibodies against B2GP1 are the main culprit in NETs formation and its release is significantly augmented by ACL, in addition, they found a positive correlation between anti-B2GP1 IgG, LAC, ACL IgG and circulating MPO-DNA complexes. The same data was supported recently by Reshetnyak et al., 2023 [20].

In all our studied subjects, and in the APLS patients’ group alone, both MPO and Histones showed significant positive correlations with PT and INR which indicates an underlying coagulopathy found due to NETs formation hand in hand with immune-dysregulation specific in APLS since these findings were not observed in the reactive or normal control groups. The same findings were supported by Bravo-Barrera et al., 2017 [19]*,* who highlighted the role of neutrophils in connecting the inflammation and coagulopathy induced by NETs formation which contributes to thrombosis (both venous and arterial) through various operations for instance binding and activating platelets, tissue factor (TF) and coagulation factor VII, which augment or initiate thrombosis. Besides, neutrophils release TF-bearing NETs which act as an activator for thrombus formation through thrombin generation. Also, Reshetnyak and Nurbaeva., 2023 [21], have also mentioned that NETs inside-constituent can suppress the fibrinolytic system and natural anticoagulants activity in the blood circulation. They also commented on the role of histones in interfering with the thrombomodulin-dependent activity of protein C. The use of anti-thrombotic treatment such as warfarin which inhibits coagulation factors synthesis especially thrombin plays an augmenting role.

There was a significant positive correlation found in all studied subjects between both MPO and Histones levels with ESR which may highlight increased NETs formation during APLS and SLE activity. Some Authors of previous research suggested that during flaring episodes of SLE activity, NETs levels are increased along with thrombotic complications furthermore, the MPO-DNA complex levels were elevated in patients with high anti-dsDNA, hypocomplementemia and glomerulonephritis indicating SLE activity [20].

Finally, upon studying the relation between NETs formation and the clinical characteristics of APLS patients, both MPO and Histones could not differentiate between 1ry and 2ry APLS which could be explained by common links in SLE and APLS pathophysiology. However, MPO and Histones were significantly higher in thrombotic APLS patients when compared with non-thrombotic ones. Besides, Histones levels were significantly increased in patients with aPL antibodies with double positivity (LAC, B2GB1or ACL) and triple positivity (LAC, B2GB1, ACL) when compared to those with single positivity (LAC only). Meanwhile, MPO level showed no significant difference among APLS patients based on their positivity for aPL antibodies. Our later findings suggest that NETs formation especially with a higher Histones level had a bad prognostic indication being associated with a more severe disease course. In concordance with our findings, Reshetnyak et al., 2023 [20] found that increased NETs formation namely MPO-DNA complex was more pronounced among thrombotic APLS patients with a poorer prognosis, such as those with multiple aPL antibodies activity and recurrent thrombosis. In addition, a large number of published researches highlighted the presence of elevated NETs in venous and arterial thrombi, lupus nephritis, immune-inflammatory mediated organ dysfunction, and pregnancy-related morbidity [19, 22].

## Conclusion

In this research, we highlighted the probable pathomechanism of NETosis in APLS and documented an increase in specific NETs markers, namely MPO and Histones, in the serum of APLS patients. These markers’ positivity was accompanied by the presence of thrombosis, coagulopathy, lower Hb levels, and disease activity. Histone levels were more indicative of the autoimmune process which is the suggested etiology of NETs activation in APLS, while MPO was more related to WBCs count and neutrophilia threshold, together proving the alliance between NETosis and the associated immune and non-immune mediated clinical manifestations of APLS.

The combined elevation of MPO and Histones at a cutoff > 2.09 for MPO and > 1.45 for Histones can be regarded as promising biomarkers for timely initiating NETs inhibitors in APLS, which is supposed to significantly lower thrombotic events, improve associated anemia and coagulopathy with better control of disease activity.

Future clinical studies and trials are needed to accurately approve the clinical application of our suggested cut-off theory as this is the first time to been postulated. Also, Further comprehension to balance the natural role of NETs activation in the host defenses and suppression/degeneration using NETs inhibitors in APLS will be essential.

## Data Availability

The datasets used and/or analyzed during the current study are available from the corresponding author on reasonable request on https://docs.google.com/document/d/1E4O7HlJ1XfPI7R0dk9nWG6XffpltkqlZ/edit. Data sharing applies to this article as datasets were generated and analyzed during the current study.

## Abbreviations

APLS: Antiphospholipid syndrome
SLE: Systemic lupus erythematosus
NETs: Neutrophil extracellular traps
aPL: Antiphospholipid antibodies
B2GP1: Anti-β2glycoprotein I antibodies
LAC: Lupus anticoagulant
ACT: Anticardiolipin antibodies
MPO: Myeloperoxidase
MPO-DNA: Myeloperoxidase–deoxyribonucleic acid complex
dRVVT: Diluted russell viper venom time
ELISA: Enzyme-linked immunosorbent assay
PLT: Platelets
Hb: Hemoglobin
